# Innovative Strategies in Precision Psychiatry: Merging Artificial Intelligence with Psychoneuroimmunology for Enhanced Mental Health Outcomes

**DOI:** 10.1192/j.eurpsy.2025.1650

**Published:** 2025-08-26

**Authors:** A. Hakimjavadi

**Affiliations:** 1 Psychology, Universitat Rovira I Virgili, Tarragona, Spain

## Abstract

**Introduction:**

The paradigms of diagnosis and evaluation in mental health are changing due to the incorporation of artificial intelligence (AI) into other fields. Understanding mental health conditions is greatly aided by insights from neuroscience, immunology, social and clinical psychology, and cultural theories. According to Martyn Evans’ commentary, interdisciplinary work generates new issues and solutions while multidisciplinary work preserves unique viewpoints. In this sense, the interdisciplinary field of psychoneuroimmunology has contributed significantly to our understanding of mental health. It has also contributed significantly to the interplay between the immune system, the endocrine system, and the nervous system. This field could rapidly emerge as a key component of integrative diagnosis and assessment.

**Objectives:**

This review emphasizes the need for comprehensive biopsychosocial assessment frameworks and the importance of harmonizing disciplines through multidisciplinary and interdisciplinary methodologies to enhance diagnostic possibilities via AI.

**Methods:**

A critical review of clinical psychology was conducted, as well as a discussion of the necessity of using integrative methodologies in order to address the interconnected nature of both medical diseases and mental disorders, in light of recent advancements in artificial intelligence.

**Results:**

It has been explored how PNI can serve as an interdisciplinary ground for cross-disciplinary dialogue and how stakeholder perspectives may resolve complexities in clinical assessment and psychiatric diagnosis through extending PNI with AI and applications. Integrating AI into PNI is crucial for revolutionizing mental health care, utilizing machine learning to consolidate diverse data streams and predict outcomes.

**Conclusions:**

Lastly, it was outlined some pragmatic frameworks for clinical assessment, taking into account time, budget constraints, and stakeholder interests. Ethical, governance, and practical challenges of AI integration are discussed. The paper proposes innovative AI-driven enhancements in psychiatric assessment, diagnosis, and treatment, fostering transformative developments in clinical psychology and promoting a humanistic approach to mental health care.

**Disclosure of Interest:**

None Declared

